# Evaluation of Pearlite Steel Thermite Weldments’ Hydrogen Degradation for Application of Additively Manufactured Crack-Resistant Material Inserts

**DOI:** 10.3390/ma19010051

**Published:** 2025-12-22

**Authors:** Michał Kawiak, Alexander I. Balitskii, Marcin A. Królikowski, Valentina O. Balitska, Jakub M. Dowejko

**Affiliations:** 1 Department of Mechanical Engineering and Mechatronics, West Pomeranian University of Technology in Szczecin, 19 Piastow Av., 70-310 Szczecin, Poland; michal.kawiak@zut.edu.pl (M.K.); marcin.krolikowski@zut.edu.pl (M.A.K.); 2 Department of Strength of the Materials and Structures in Hydrogen-Containing Environments, Karpenko Physico-Mechanical Institute, National Academy of Sciences of Ukraine, 5 Naukova Str., 79601 Lviv, Ukraine; 3 Department of Physics and Chemistry of Combustion, Lviv State University of Life Safety, 35 Kleparivska, 79000 Lviv, Ukraine; vbalitska@yahoo.com; 4 Institute of Management, University of Szczecin, Cukrowa 8, 71-004 Szczecin, Poland

**Keywords:** hydrogen, fracture toughness, brittle cracking, thermite welding, tram rails

## Abstract

Comprehensive investigations of the serviceability of pearlite (R260) steel have been performed and, especially, of the serviceability of their welded joints (WJ) during long-term operation in hydrogen-containing environments for application in additive manufacturing technology. It is important to estimate the durability of these steels and their WJ in hydrogen and develop the procedures of analysis of the influence of hydrogen during long-term operation. It has been experimentally observed that hydrogen absorbed (0.4 … 0.8 ppm) by the pearlite (R260) steel while welding, and subsequent operation thereof, exercises considerable influence on fatigue and brittle fractures of the constructions from which they are manufactured. Accordingly, in hydrogen-saturated (up to 4.7 ppm) specimens, the desired fatigue crack can be obtained at a considerably lower number of cycles of the same dynamic load than in non-hydrogenated ones. Increased hydrogen content can also affect crack propagation. Tests have shown that critical fracture occurs faster in hydrogenated specimens (46.6 MPa m^0.5^) than in non-hydrogenated ones. Also, hydrogenated specimens exhibit lower fracture toughness than their non-hydrogenated counterparts. Finally, it has been demonstrated that the fracture toughness of specimens taken from rail negligibly (49.7 … 50.7 MPa m^0.5^) depend on their orientation (L–S or S–L).

## 1. Introduction

Numerous instances of cracking that occur during welding of rails as well as their further operation indicate that the problem is still common and current. On the other hand, as far as tests required for tram rails (made from perlite steels) are concerned, the EN 13674-1+A1 [1,2,3,4,5,6,7,8,9,10,11,12,13,14] standard does not provide for brittle cracking tests. Lacking proper knowledge, one cannot predict the behaviour of rails operating in each environment and under dynamic loading and ambient temperatures.

Therefore, conducting brittle cracking tests for such rails proves relevant, especially whenever the safety of passengers is involved. Despite considerable development in modern technology in the thermite welding of rails (based on the thermodynamic principles of the thermite reaction and such characteristics as adiabatic temperatures and heat realisation), especially in terms of rail material, thermite mixtures, equipment, and weld control [3,4], the instances of rail cracking still occur in the course of welding or rail operation. When an accident happened—a rail cracked under a passing tram and a gap in the track appeared—rail welded joints were tested on modernised and newly built tram lines. Altogether, several hundreds of cracks, varying in length, were discovered. This fact inspired the present authors to conduct research on the causes of cracking of thermite-welded tram rails. According to the authors’ own research, hydrogen content in the cracked rail near the crack tip equals 0.80–1.37 ppm, while, according to the certificates, the content equals up to 2.0 ppm, depending on melt number. It can happen that a rail cracks under such loading. Fortunately, cases of rail fracture resulting in accidents involving people are very rare [3,4]. Thorough examinations are conducted following such events to determine what caused the accident to happen and what needs to be done to prevent it from happening again. Apart from loads, other factors conducive to railway and tram rail cracking include the properties of rails and the environment in which they are in operation. This article presents the results of tests for resistance to brittle cracking of tram rail [3,4,5].

The scope of railway and tram rail tests that are required before rails can be installed in a track is stipulated in the EN 13674-1+A1 [1] and EN 14811 [2] standards.

**Figure 1:**
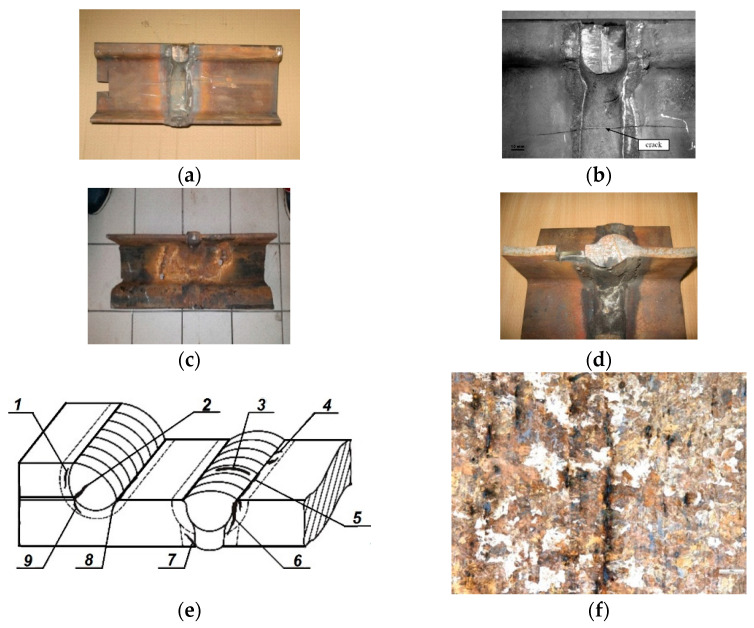
Common view with different cross-sections of thermite weldments (**a**–**d**), possible crack initiation (1—circumferential in HAZ, between tram rail and thermite, 2—radial in WM, 3—circumferential in WM, 4—radial in HAZ, 5—axial in HAZ, 6—initiated from top of WJ in plate weldment, 7—initiated from upper of WJ in plate weldment, 8—axial in angle weldment HAZ, 9—circumferential in angle weldment HAZ) in the course of thermite welding (**e**) and microstructure of a tram rail, cracked during thermite welding (×400) (**f**).

In most cases, in the initial phase, the cracks were parallel to the direction of rolling (Figure 1) and propagated in various directions during further operation of the rails.

The aim of the present work is to establish the regularities of the influence of “metallurgical” hydrogen on the mechanical properties of materials and their WJ and develop approaches for the evaluation of the serviceability of these materials and joints during long-term operation in structural elements in contact with hydrogen containing environments in railway system, which will be useful for application of additively manufactured crack-resistant material inserts.

The work is devoted to the investigation of hydrogen influence on the structural material properties’ degradation using modern fracture mechanics approaches, partially on the fracture toughness (K_IC_), which now is a new materials characteristic in the circumstance of the realisation of a plane stressed state or elastic deformation conditions [9,14]. The resistance of rails to brittle cracking can decline during their operation due to acid rains, salts, alcohols, acetates, and formats that contain—among many other elements—hydrogen and are used to remove icing from roadways in the wintertime. Such an interaction can be caused by the increase in hydrogen in rail material. The results of tests of fracture toughness of hydrogenated and non-hydrogenated specimens obtained from tram rails have been discussed.

Additive manufacturing (AM) (or 3D printing) was applied to create the final tram rail steels and their WJ, which has been used in the construction and transport industry [6]. Due to specific geometry of layer by layer in AM technologies, “printed” hydrogen-resistant (layer by layer) material [7] is of significant interest in the construction and transport sectors. Fusion-based AM, such as thermite, wire arc additive manufacturing (WAAM), and laser powder bed fusion (SLM and SLS), will be used soon due to directed energy deposition phenomena. The mechanical performance of low-alloy steel has shown increased tensile strengths after AM processing compared with conventionally manufactured (CM) [6].

Investigation into the impact of hydrogen embrittlement (HE) on the mechanical properties and crack resistance of AM structural materials is of utmost importance for industries utilising these steels. The unique characteristics of AM processes may introduce additional factors that affect the complex hydrogen–materials interactions and HE, such as the hydrogen accumulation at phase interfaces and local reaching of the critical hydrogen concentration. The specific layered microstructure [7] of AM and interfaces between phases in the microstructure present crucial factors that influence HE. Low-alloyed steels, such as R260, follow a distinct mechanism where pearlite transformation predominantly governs HE. The effect of HE in additively manufactured steel seems to be less pronounced compared to CM metals [8]. Future studies should aim to develop a more comprehensive understanding of complex and multiple HE effects on the mechanical properties of AM manufactured steels and HE-assisted fatigue [8].

## 2. Materials and Methods

**Figure 2:**
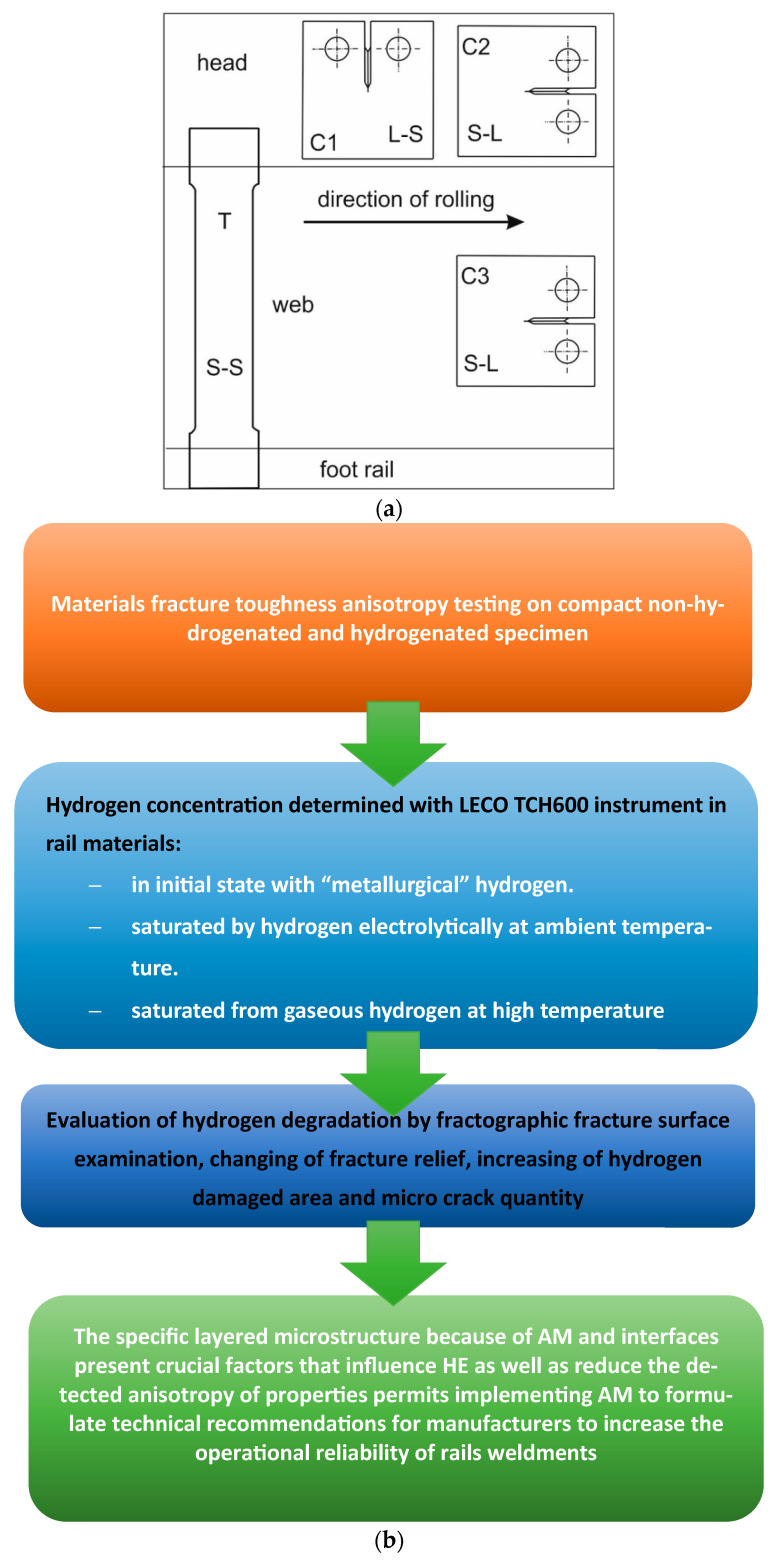
Places of sampling of compact specimens C used in fracture toughness tests and specimens with a rectangular cross-section T used in tensile tests (**a**); experimental flowchart (schematic diagram) of the applied methodology (**b**).

Tests were conducted on a notched Vignola tram rail made from R260 steel. Temporary values of stress intensity factor were determined with the use of compact specimens, taken from the rail head and web [8,9,10,11,12,13,14,15,16,17,18,19,20,21]. Specimens had an L–S or an S–L orientation. To determine the influence of hydrogen on the anisotropy in propagation of fatigue crack and brittle cracking, some of the specimens underwent electrochemical hydrogenation. A fractographic examination of the fractures was also conducted. The article [3] features the results of tensile strength tests and fracture toughness tests as well as the outcomes of the examination of microstructure and chemical composition of tram rails made from R260 steel that cracked during thermite welding. As far as fracture toughness tests are concerned, this work presents only the values of stress intensity factors determined by compact specimens taken from the rail head in which the plane of the fracture was perpendicular to the direction of rolling of the rail. In the present article, the scope of testing was extended to discover the relationship between increased amount of hydrogen and quicker crack propagation. Fracture toughness tests of tram rails made from R260 steel were conducted on INSTRON 8850 machine (INSTRON, Norwood, MA, United States), in accordance with the ASTM 399-19 standard [4]. EN 13674-1+A1 and EN 14811 standards [1,2] does not provide for brittle cracking tests for tram rails; therefore, the shapes and sizes of specimens were selected following recommendations contained in EN 14811 standards [2]. The articles [5,6,7,8,9,10,11,12,13,14,15,16,17,18,19,20] showed that, while structural materials are in operation in a corrosive environment, electrochemical corrosion takes place. Due to cathode reaction, the process involves hydrogen release and transfer of metal ions into the solution. The two processes, i.e., corrosion and hydrogenation, take place simultaneously [12]. According to [13], the corrosion process affecting welded joints occurs at different speeds in different parts of the joint (weld, fusion line, and heat-affected zone). In light of the findings above and the article [3], it is very important to examine the influence of hydrogen concentration on the parameters of the process of crack propagation. Following the standard [2], the first set of compact specimens C1, in which the orientation of the fracture plane was L–S, was taken from the rail head. Places of sampling and specimen orientations are shown in Figure 2. In addition (as a comparison) [1], tests were also conducted on compact C2 and C3 specimens with an S–L orientation. In these specimens, the orientation of the fractured planes is the same as that of most rail cracks. The C2 specimens were taken from the rail head, and the C3 specimens from the rail web. A batch of C3 specimens was divided into two sets (3 pieces each). Prior to fracture toughness tests, three specimens underwent hydrogenation. The procedure was conducted with an electrolytic method and by means of hydrogen cathodic saturation in a 26% solution of H_2_SO_4_ acid. The voltage and density of direct current were U = 2.6–2.7 V and I = 0.05 A/cm^2^, respectively. Hydrogenation time t = 2 h. The process of electrochemical hydrogenation differs from the process of hydrogenation in a gaseous hydrogen environment and pore formation [17], both in terms of the amount of absorbed hydrogen and local concentration in particular areas of the tested specimens. To review the effects of both processes, selected specimens had been hydrogenated 2 h in a gaseous hydrogen environment at an elevated temperature of 623 K and the pressure of 10 MPa (no tempering effect in pearlitic steel was observed during that time). The choice of this temperature and holding time for 2 h during the carburisation process takes into account the short-term effect of the combustion temperature of the thermite mixture within 2300–2600 °C and the inertia in time of heat transfer processes, which allows us to offer the obtained results for adequate comparability with real carburisation processes that occur during thermite welding, which increase the saturation by “metallurgical hydrogen” and its accelerated high temperature diffusion.

In contrast to cathodic hydrogen saturation, which leads to increased hydrogen concentration in sub surface layers, such a regime ensures homogeneous hydrogen concentration in the whole material volume. Afterwards, hydrogen concentration was measured for both sets of specimens, i.e., those hydrogenated with the use of either the first or the second method. Hydrogen concentration was determined with LECO TCH600 instrument. In the specimens taken from the rail head, fatigue cracks at the bottom of the notch were obtained with sinusoidal alternating tensile force that had the following parameters: amplitude Fa = 6 kN, stress ratio R = 0.1, and frequency f = 50 Hz; in the specimens taken from the rail web, the cracks were produced with a force with the following parameters: Fa = 2.5 kN, R = 0.1, and f = 40 Hz.

**Figure 3:**
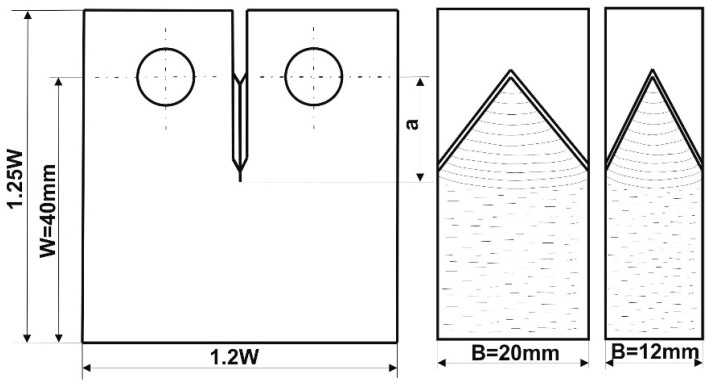
Basic dimensions of a compact specimen.

In order to obtain a plane state of strain in the crack tip, the specimen ligament size (W-a) (Figure 3) must not be less than 2.5 (K_Ic_/σ_YS_)^2^, where K_Ic_—fracture toughness and σ_YS_—yield strength (0.2%).

Due to the shape and size of the rail head, it was possible to cut out compact specimens of maximum dimension values W = 40 mm and B = 20 mm. The dimensions of specimens taken from the rail web were the following: W = 40 mm and B = 12 mm (nominal thickness of the web t = 12 mm).

To estimate the validity of the K_Ic_ test, it is necessary to know the yield point σ_YS_ of the tested material in each environment and at a given temperature. Tensile tests were conducted on specimens with a circular cross-section, taken from the rail head parallelly to the direction of rolling, and specimens with a rectangular cross-section T (S-S) (Figure 2).

Tensile tests were conducted in the same conditions as fracture toughness tests, i.e., with INSTRON 8850 machines in a room atmosphere and temperature. A feature of the methodology of the proposed experiment is, given the significant content of metallurgical hydrogen in rail steels in the delivered state and its additional accumulation during the thermite welding process and long-term operation in hydrogen-containing environments, to investigate the process of hydrogen degradation of these structural materials and consider the prospects for applying incremental technologies to materials with increased resistance to hydrogen cracking for use in the repair of damaged sections of existing railways.

## 3. Results and Discussion

### 3.1. Tensile and Chemical Composition Test

The results of tensile tests of specimens with circular and rectangular cross-sections are given in Table 1. For all the four specimens, the tensile strength R_m_ and elongation A fulfil the requirements of the EN 13674-1+A1standard (R_m min_ = 880 MPa; A_min_ = 10%). The average value of R_m_ for the specimens taken from the rail head is 2% higher than the average value of R_m_ for the specimens taken from the rail web. Therefore, it can be presumed that the tensile strength of these specimens is practically the same. The differences in the value of the yield point σ_YS_ are much bigger. The average value of σ_YS_ of the specimens taken from the web is 20% higher than the average value of σ_YS_ of the specimens taken from the head. This fact can be explained by the fact that the cold work of the rail web is greater than that of the rail head. The chemical composition (C, Mn, Si, P, S, and Cr) of R260 rail steel was determined with the use of an optical spectrometer with GDOES. The results of the tests are given in Table 2.

### 3.2. Fracture Toughness

It has been shown in [1,2,3,4,5,6] that, currently, fracture toughness tests prove insufficient. This fact is especially prominent in the case of tram rails, since it is not possible to determine their resistance to brittle cracking based on the recommended tests. The authors considered it worthwhile to determine the resistance to brittle cracking of tram rails. It was taken into consideration that trams going on a newly built track in Szczecin move at a high speed, i.e., 70 km/h, that numerous rail cracks were found on the track, and that the removal of the cracks in question required a lot of time and resources.

**Figure 4:**
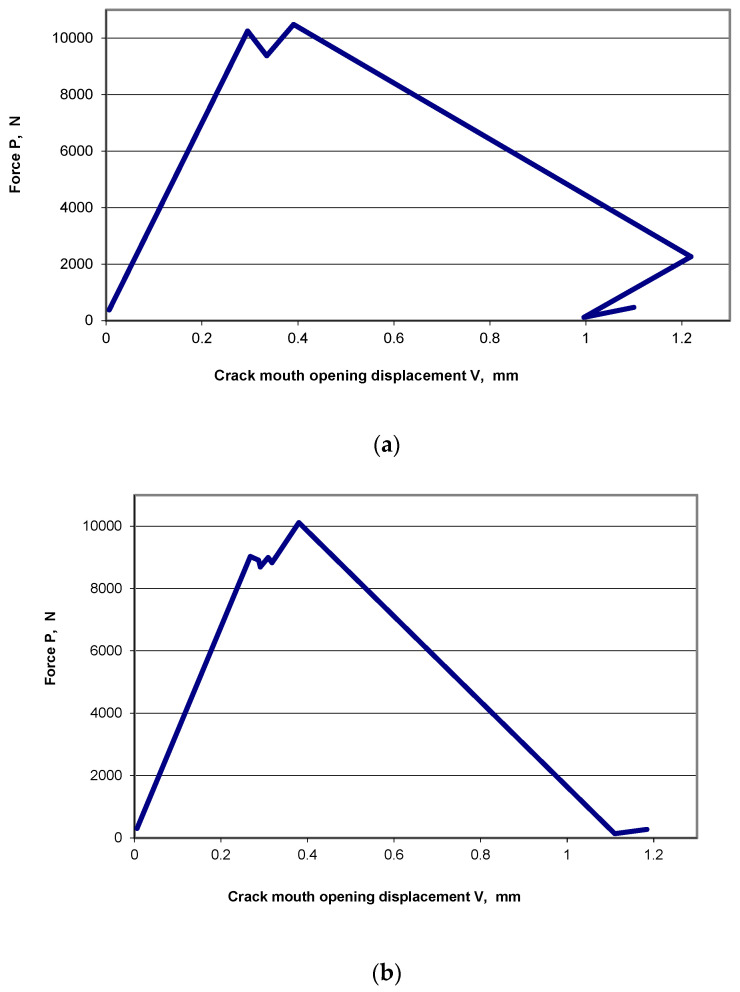
Tensile test diagrams for compact specimens of an S–L orientation, taken from the rail web: (**a**) non-hydrogenated specimen; (**b**) hydrogenated specimen.

Fatigue cracks of a desired length a = 22 mm for the specimens taken from the rail head and having L–S and S–L orientations were obtained with an almost identical average number of fatigue load cycles (n_L-S_ = 132,600; n_S-L_ = 132,500). A considerable difference was observed, however, in the case of the specimens taken from the rail web with an S–L orientation, both the non-hydrogenated and hydrogenated ones. The desired crack was obtained with an average number of fatigue load cycles amounting to n = 85,800 for non-hydrogenated specimens and n_H_ = 61,900 for hydrogenated specimens. The difference is thus 28%, which indicates a considerable influence of hydrogen absorbed by the material on the formation of fatigue fracture. Comparing the diagrams of tensile tests on compact specimens, non-hydrogenated and hydrogenated ones, it can be observed that hydrogen affects also the propagation of the crack. The process consists of two phases: subcritical crack growth—the crack propagates relatively slowly during a temporary decrease and then increase in the load; critical crack growth—the propagation of the crack can no longer be controlled and does not require increasing the load. A tensile test diagram for a non-hydrogenated specimen (Figure 4a) shows that, in the subcritical cracking phase, one cycle of increasing and decreasing of the load was registered, while a diagram for the hydrogenated specimen (Figure 4b) shows that there were a few such cycles. It also needs to be emphasised that critical cracking of hydrogenated specimens occurs at a higher speed than that of non-hydrogenated specimens.

The results of fracture toughness tests are given in Table 3. For each temporary value of the stress intensity factor K_Q_, verification calculations were made, following condition W-a ≥ 2.5(K_Ic_/σ_ys_)^2^ concerning the occurrence of plane state of strain in the crack tip. It turned out that the results marked with a superscript do not fulfil this condition. In general, condition W-a ≥ 2.5(K_Ic_/σ_ys_)^2^ was not fulfilled for eight specimens taken from the rail head. The specimen ligament size (W-a) was lower than the required size that would ensure plane state of strain in the crack tip.

For two specimens taken from the rail head and having L–S and S–L orientations as well as for all specimens taken from the rail web and having an S–L orientation, one can find plane state of strain in the crack tip (K_Q_ without the superscript). For these specimens K_Ic_ = K_Q_. The third column (Table 3) features arithmetic average values of the K_I_ stress intensity factor of the specimens with L–S (K_I_ = 49.7 MPa m^0.5^) and S–L (K_I_ = 50.6 MPa m^0.5^) orientations taken from the rail head.

The two values do not really differ. It can be noticed that, in the head area of the tested rail, fracture toughness is not dependant on specimen orientation (L–S or S–L). As was mentioned above, the condition W-a ≥ 2.5 (K_Ic_/σ_ys_)^2^ was not fulfilled for eight specimens taken from the rail head. This means that that the internal part of the specimen that adjoins the crack tip is in plane state of strain, and the part adjoining the external surfaces is in plane state of stress. In such a case K_I_ = K_Q_. Fracture toughness K_I,_ measured in partial plane state of strain and partial plane state of stress is of significance only for the specimen thickness considered in the tests.

Increased hydrogen concentration in local volumes of the construction material of tram rails can lead to a high increase in the stress around the crack tip. Such an observation is consistent with the results obtained by the authors of the papers [10,11]. The article [11] states that hydrogen-induced stress σ_ad_ increases linearly, along with the rise in hydrogen concentration C_o_, σ_ad_ = −14.1 + 3.89 C_o_, while hydrogen-induced cracking threshold stress intensity σ_HIC_ decreases linearly along with the rise in the logarithm of concentration lnC_o,_ σ_HIC_ = 669–124 lnC_o_.

For example, for specimens taken from the tram rail, σ_ad_ = 43.1 MPa and σ_HIC_ = 335.7 MPa for C_o_ = 14.7 ppm (Table 3). A significant increase in the stress around the crack tip [10,11] may result in a substantial drop in the value of stress intensity factor and drastic crack propagation. We are of the opinion that such a phenomenon occurred during tram rail cracking on a section of a fast tram line in Szczecin. The areas of brittle cracking that could be observed on the surfaces of the cracks in the course of fractographic examination also confirm the hypothesis that hydrogen bears significant influence on tram rail cracking. The view that the hydrogen that comes from anti-icers used to remove icing from tram tracks in winter exerts a considerable influence on tram rail cracking is fully consistent with the papers [10,11].

Arithmetic average values of the K_Ic_ factor of the specimens taken from the rail web are given in the third column of Table 3. For non-hydrogenated specimens, the average value K_Ic_ = 50.7 MPa m^0.5^ and, for hydrogenated specimens, K_Ic_ = 46.6 MPa m^0.5^. As a result of electrochemical hydrogenation, the value of the K_Ic_ factor is, on average, 4.1 MPa m^0.5^ lower than the value of K_Ic_ for non-hydrogenated specimens.

The SIF factor K_Ic_ of non-hydrogenated specimens taken from the rail web and having the S–L orientation is practically the same as the K_I_ factor of non-hydrogenated specimens taken from the rail head and having the same orientation (K_Ic_ = 50.7 MPa m^0.5^, K_I_ = 50.6 MPa m^0.5^, Table 3). Looking only at the diagrams illustrating the influence of the yield point on fracture toughness, the factor K_Ic_ should be smaller than K_I_. The yield point of the web material is higher than the yield point of the rail head (Table 1). It means that the brittleness of the web is higher than that of the head, which is reflected in the drop in the K_I_ value. In the case of the tested specimens, it is not only the influence of the yield point on specimen brittleness per se that must be considered. The influence of specimen thickness on the value of the SIF also needs to be considered. The minimal thickness of a specimen that affects the plane state of strain in the crack tip B_C_ = 2.5 (K_Q_/σ_ys_)^2^. Based on the relation between the K_Q_ factor and thickness B of a specimen, it can be stated that, the lower the value of B gets in relation to B_C_, the higher the value of the K_Q_ factor gets in a nonlinear way. Therefore, the K_Ic_ ≈ K_I_ inequality presented above is correct. In the case of the specimens taken from the rail head and web, the influence of specimen thickness on the stress intensity factor counterbalances the influence of the yield point.

It must be taken into account that, all over the world, rail roads are traditionally placed above the ground level. This is, however, not the case with city tram rails. Therefore, apart from undergoing classic corrosion in, for example, chloride solutions [12] (which are formed in a city road environment in the wintertime and during other seasons), city tram rails are often submerged in rain solution (and other aggressive chemicals, potentially hydrogenated). This study thus constitutes a new approach to experimentally established hydrogen embrittlement of city tram rails (with a fixed concentration of “non-metallurgical” hydrogen) and to the comparison of the influence of subsurface and volume hydrogen concentration on the process in question.

At certain specimen thickness, K_c_ = K_Ic_. In such cases, the fracture is flat and brittle, without signs of plastic strain on the lateral surfaces of the specimens, as has been also established by tests conducted with various experimental methods [9,10,11,12,13]. At maximum hydrogen embrittlement, the K_c_ coefficient is almost independent from specimen thickness. This fact highlights the practical value of the K_IcH_ for hydrogenated steels. When the hydrogen content is lower than the critical content, the influence of specimen thickness and hydrogen content on the K_c_ coefficient is clearly noticeable. For the evaluation of steel crack resistance in a gaseous hydrogen environment to be correct, it is important to determine the hydrogen content at which plane state of strain takes place. Hydrogen increases the susceptibility to brittle cracking, and plane state of strain takes place at much lower specimen thickness. The considerable influence of hydrogen on the size of plastic zone, proportionally dependent on the square of the K_c_ coefficient, needs to be emphasised [13]. Hydrogen also determines the division of strains into elastic and plastic before crack front and influences the type of destruction and the remaining service life. It is known that, in case of big specimens, the dimensions of the plastic zone are small, as compared to the thickness thereof and the length of fatigue cracks. The influence of high hydrogen concentration on steel brittleness becomes more clearly visible in the case of tests on specimens with lower thicknesses and higher quotients of plastic zone dimensions and higher specimen thicknesses. It means that the influence of high amount of hydrogen on susceptibility to brittle cracking is stronger in the case of specimens with plane state of stress than in the case of specimens in plane state of deformation.

### 3.3. Fractographic Examination

**Figure 5:**
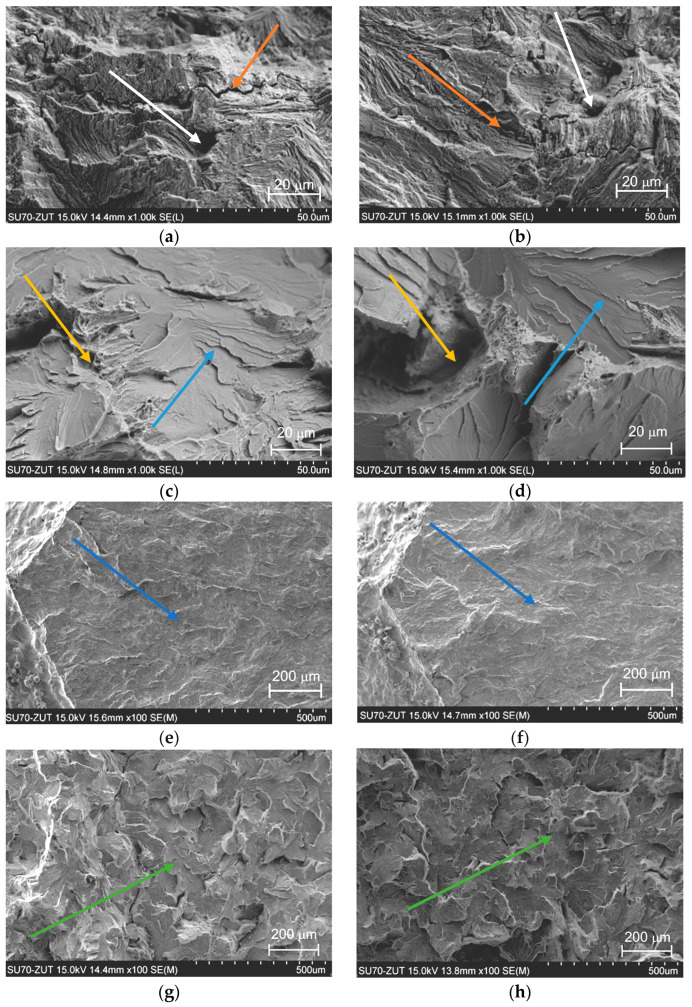
Fracture surfaces around fatigue crack: (**a**,**b**)—non-hydrogenated specimen, (**c**,**d**)—hydrogenated specimen, around pores and brittle crack initiation in the specimen in initial state with “metallurgical” hydrogen (**e**), specimens after electrochemical hydrogen saturation (**f**) and high temperature gaseous hydrogenation (**g**,**h**). Map of arrow colours: round dimple, combine round and parabolic dimple, cleavage, pores, microcracks, flakes, and surfaces of smooth splitting.

The examination of fractures was conducted with an electron microscope, Hitachi SU-70 (Hitachi High-Tech Corporation, Ichige, Hitachinaka, Japan). The mechanism of transcrystalline cracking that goes through the grains of the material, perlite in this case, can be observed on the fractures of specimens taken from the rail web (Figure 5). The images of fatigue and brittle fractures differ to a large degree.

If focusing on the interface between the fatigue pre-crack region and the propagation zone occurring during the fracture toughness test, we have observed the difference between the non-hydrogenated and hydrogen-saturated specimens.

The cracks that can be observed in Figure 5b,d were found in the areas where the content of non-metallic inclusions (mainly sulphur) was higher.

The hydrogen left in the steel can considerably lower the steel’s resistance to brittle cracking and it is sufficient for increasing by 10 … 100 times near the crack tip [8,9,10,11,12,13].

Figure 5 clearly indicates the fracture modes. Arrow colours identify specific fracture mechanisms: round dimple, parabolic dimple, cleavage, pores, microcracks, flakes, and surfaces of smooth splitting with support in the next corresponding analysis.

In both cases—electrochemical hydrogenation and hydrogenation in a gas environment—microcracks appeared on the surface of the specimens [14,15,16,17,18,19]. The formation of surface cracks in the presence of hydrogen occurred in a manner typical of a pearlitic structure. When strain speed had fallen and hydrogen concentration had increased, the number and depth of cracks in the specimens rose.

The most universal microfractographic element of the ductile fracture surface is round or parabolic dimples (Figure 5a,b). When analysing the main modes of ductile fracture, normal detachment and shear failure are possible.

Viscous normal separation occurs as a result of intense plastic deformation of the material under the influence of uniform tensile stresses dominating in one direction. This leads to the formation of microvoids (Figure 5c,d), which are elongated in the direction of maximum tensile deformation.

The voids themselves arise as a result of incompatible deformation of individual structural components (for example, hard particles with an underlying soft matrix), as well as due to possible delamination (fibrousness) associated with the metallurgical anisotropy of the material (Figure 5e,f).

Such axially elongated voids eventually merge due to the rupture of the material of the partitions between the voids, which gradually become thinner. As a result of such destruction, equiaxed dimples are formed (Figure 5a,b) and traces of inclusions or precipitates are observed on their surface, from which the nucleation of microvoids began.

In the case of intense shear plastic deformation, which is realised at a high level of tangential stresses, the formation and growth of microvoids (Figure 5c,d) occurs in the plane of the voids themselves, which is constantly thinning during the deformation process. The elongated microvoids thus approach each other and finally merge circumferentially by breaking the membranes in the void layer. The dimples formed on the surface have an elongated parabolic shape (Figure 5a,b).

The effect of hydrogen is immediate. This is explained by the chemisorption of hydrogen on iron, which occurs instantaneously, which corresponds to the inertia-free nature of crack propagation. Hydrogen changes the nature of the fracture, the fracture becomes intercrystalline, and local sub-microcracks and chips are observed along the planes of smooth cleavage (Figure 5e,f) due to the more frequent occurrence of secondary cracks (Figure 5c,d).

Track marks are also of hydrogen origin. Initially adsorbed on juvenile surfaces near the crack tip, hydrogen penetrates into the subsurface high-stress volumes (Figure 5g,h) (a loose scaly fracture surface is formed) and, reaching a certain concentration, is released through the formation of microvoids.

Based on the results obtained in situ and the results of structural and functional material degradation with the use of SEM and TEM microscopes [21,22,23,24,25], it can be established that the influence of absorbed hydrogen directly affects crack propagation and fracture surface in a way that is characteristic for brittle fracture [26,27,28,29,30,31].

Emission and greater mobility of dislocation, resultant from the influence of hydrogen, are proportional to the increase in the external load on the bent specimens [11]. It means that hydrogen affects emission and intensification of the dislocation movement in the same way as external load. During conduction fractographic examination, it has been established that hydrogen exerts influence on tram rail fracture.

In the microstructure of the fractures of hydrogenated specimens, the mechanism of brittle shear fracturing prevails in the area where surface cracks are present (Figure 5c). On the surfaces of the cracks, there are areas featuring numerous secondary cracks, partially on the grain boundaries (Figure 5d). The characteristic features of hydrogen-initiated cracks can be observed in many other steels as well [1,5,11,14,15], in contrast to the cracks that appear in air environment, where usually only one direction of cracking can be observed (Figure 5b). The microstructure of the crack around near-surface ductile cracks is usually an IG crack (Figure 5b). It is due to the precipitation of carbides on grain boundaries. Fractographic examination of the cracks around the initial fatigue crack front indicates the character of destruction in this zone was typical of plastic materials: dimple microreliefs and transcrystalline spread of the crack with separate IG cracks (Figure 5a). On the surface of crack of hydrogenated specimens, there prevail areas of IG cracking, quasi-cleavage facets with hackles along the boundary’s carbide matrix and slip-band separations, and flat and 3D facets. In all cases, structural defects are conducive to crack nucleation and spread in the case of hydrogen presence [1,5,11,14,15].

Hydrogen is responsible for uneven crack propagation near the threshold region and lowering of threshold values of crack resistance [32,33,34,35,36,37,38,39,40,41,42,43,44,45,46,47,48], induces accelerated crack growth [18], co-participates in embrittlement mechanism via mobile atoms [49,50,51,52,53,54,55,56,57,58,59,60,61], and alters mechanical parameters, i.e., Young’s modulus [19], in many steels and iron-based alloys.

The initial hydrogen microcracks can be detected in the crack tip areas (Figure 5d), where the content of non-metallic inclusions (mainly sulphur) was higher (due to EDX spectra). Microcracks appear on the same surface of specimens, hydrogenated from hydrogen contained environment (Figure 5). The formation of surface cracks in the presence of hydrogen occurs in a manner typical of a pearlitic structure. When the strain speed falls and hydrogen concentration near the crack tip increases, the cracks’ number and depth in the specimens rise [62,63,64,65,66,67,68,69,70,71]. In addition, there are few micro areas of ductile cracking. The formation of such areas begins with the emergence of micro-voids originating from the intensive plastic strain. As they grow, microvoid coalescence dimples (MVC) blend, permitting the formation of the main crack, as structures of densely distributed indentations of different shapes, called dimples, in the relief. The transformation of fracture mechanisms has been established from viscous trans granular with elements of storm microrelief in neutral environments to brittle quasi-detachment in the presence of hydrogen.

In contrast to the case of applying thermite weldments AM or 3D printing to create the final tram rail steels and their WJ, due to the specific geometry of layer by layer in AM technologies, “printed” hydrogen-resistant (layer by layer) material will permit reduced crack initiation in the circumferential in HAZ direction between tram rail and thermite. Due to layered structures, cracking can be stopped in the axial in HAZ and initiation from the top of WJ or from the upper WJ in plate weldment. Axial angle weldment HAZ and circumferential angle weldment HAZ [6] during AM or 3D printing will improve the microstructure of a tram rail and prevent hydrogen-initiated cracking.

The obtained results are the base for the introduction of additive manufacturing, which can be applied to create the final tram rail steels and their WJ, which has been used in the construction and transport industry. Due to the specific geometry of layer by layer in AM technologies, “printed” hydrogen-resistant (layer by layer) material is of significant interest in the construction and transport sectors. Investigation into the impact of hydrogen embrittlement on the mechanical properties and crack resistance of AM structural materials is of utmost importance for industries utilising these steels. The unique characteristics of AM processes may introduce additional factors that affect the complex hydrogen–materials interactions and HE. The specific layered microstructure of AM and interfaces between phases in the microstructure present crucial factors that influence HE.

Confirmation of the embrittlement effect of metallurgical hydrogen, electrolytic hydrogenation, and the effect of gaseous hydrogen at high temperatures is show in Figure 5 with microstructural images, fracture morphology, and hydrogen-stimulated pore formation, which allows us to thoroughly confirm and discuss the experimental results of the study of fracture toughness behaviour under the specified conditions.

## 4. Conclusions

- Hydrogen absorbed by tram rail in the process of thermite welding as well as due to embrittlement of welded joints of tram rails in city environments exercises a considerable influence on the formation of fatigue and brittle fractures. It has been experimentally observed that “metallurgical” hydrogen absorbed (0.4 … 0.7 ppm) by the rails of a high-speed city tram while welding, and subsequent operation thereof, exercises considerable influence on fatigue and brittle fractures of pearlitic (R260) steel from which the rails are manufactured.
- Increased hydrogen content affects crack propagation: critical fracture occurs faster in hydrogenated specimens (46.6 MPa m^0.5^) than in non-hydrogenated ones. Hydrogenated specimens exhibit lower fracture toughness than their non-hydrogenated counterparts. It has been demonstrated that the fracture toughness of specimens taken from rail negligibly (49.7 … 50.7 MPa m^0.5^) depend on their orientation.
- Additive manufacturing can be applied to create the final tram rail steels and their WJ, which has been used in the construction and transport industry. The unique characteristics of AM processes may introduce additional factors that affect the complex hydrogen–materials interactions and HE. The specific layered microstructure of AM and interfaces between phases in the microstructure present crucial factors that influence HE as well as reduce the detected anisotropy of properties by implementing AM, which permits us to formulate technical recommendations for manufacturers to increase the operational reliability of rails.

## Figures and Tables

**Table 1:** Results of tensile tests conducted on the specimens taken from a tram rail made from R260 steel.

Cross-Section/Orientation/ Place of Sampling	Specimen Number	Yield Strength σ_YS_ [MPa]	Ultimate Tensile Strength R_m_ [MPa]	Elongation A [%]	Reduction Z [%]
circular L–L head	1	548	947	12.8	20.5
	2	556	957	13.8	23.4
rectangular S–S web	3	658	932	11.6	23.9
	4	663	933	11.8	23.7

**Table 2:** Chemical composition of R260 steel [%].

Test/ Standard	C	Mn	Si	P	S	Cr
test	0.630	0.868	0.306	0.013	0.019	0.023
EN 14811	0.60–0.82	0.65–1.25	0.13–0.60	max 0.03	max 0.03	max 0.15

**Table 3:** Results of fracture toughness tests conducted on the specimens taken from a tram rail made from R260 steel.

Place of Sampling, Specimen Orientation, Non-Hydrogenated (in Initial State) or Hydrogenated Specimen	Temporary Value of K_Q_ [MPa m^0.5^]					Arithmetic Average Value K_Ic_ or K_I_ [MPa m^0.5^]	Hydrogen Concentration C_H_ [ppm]
	Specimen Number						
	1	2	3	4	5		
head, L–S, non-hydrogenated	46.9	47.5 *	48.1 *	49.2 *	54.2 *	49.7 *	0.4
head, S–L, non-hydrogenated	48.9 *	53.3 *	46.0	51.2 *	49.2 *	50.6 *	1.1
web, S–L, non-hydrogenated	54.0	49.8	48.4	-	-	50.7	0.8
web, S–L, hydrogenated	45.9	47.1	46.9	-	-	46.6	14.7

Note: specimens whose K_Q_ factor values are marked with a superscript in the form of * do not fulfil the condition of plane state of strain in the crack tip.

## Data Availability

The original contributions presented in this study are included in the article. Further inquiries can be directed to the corresponding author.

## Nomenclature

C_H_	hydrogen concentration
R_m_	ultimate tensile strength (UTS)
σ_YS_	yield strength (YS)
A	elongation
Z	reduction in area
a	crack length
K_c_	plane-stress fracture toughness
K_IC_	plane-strain fracture toughness, MPa·m^½^
E	Young’s modulus
wppm	weight parts per millions
HE	hydrogen embrittlement phenomena
HELP	hydrogen-enhanced localized plasticity mechanism
HEDE	hydrogen-enhanced decohesion mechanism
MVC	microvoid coalescence, dimples
TrG	transgranular
QC	quasi-cleavage
IG	intergranular
WJ	welded joints
WM	weld metal
HAZ	heat-affected zone
BM	base metal
